# Tuning the Charge Transfer in MWCNTs via the Incorporation of ZnONPs and AgNPs: The Role of Carbon Binding with ZnO/Ag Heterostructures in Reactive Species Formation

**DOI:** 10.3390/nano14181517

**Published:** 2024-09-18

**Authors:** Ismael Gamiño-Barocio, Eric Fernando Vázquez-Vázquez, Yazmín Mariela Hernández-Rodríguez, Oscar Eduardo Cigarroa-Mayorga

**Affiliations:** 1 Department of Advanced Technologies, UPIITA-Instituto Politécnico Nacional, Av. IPN 2580, Mexico City 07340, Mexico; jgaminob1600@alumno.ipn.mx; 2 CINVESTAV-Instituto Politécnico Nacional, Av. IPN, 2508, Mexico City 07360, Mexico; fernando.vazquezv@cinvestav.mx

**Keywords:** silver nanoparticles, zinc oxide–silver heterostructures, ultrasonic-assisted synthesis, methylene blue, nanocomposite materials

## Abstract

In this research, multi-walled carbon nanotubes (MWCNTs) were decorated with two kinds of nanostructures, (1) silver nanoparticles (AgNPs) and (2) zinc oxide–silver nano-heterostructures (ZnO/Ag-NHs), via an accessible chemical coprecipitation method assisted with ultrasonic radiation. The high-resolution transmission electron microscopy analysis demonstrated the successful decoration of MWCNTs with the nanostructures with a diameter size of 11 nm ± 2 nm and 46 nm ± 5 nm for the AgNPs and the ZnO/Ag-NHs, respectively. The reactive species were promoted in an aqueous medium assisted with UV irradiation on the functionalized MWCNT. UV-Vis spectroscopy demonstrated that production of the reactive species density increased 4.07 times, promoted by the single MWCNT after the functionalization. X-ray photoelectron spectroscopy showed that Sp^2^ hybridization in carbon atoms of MWCNTs participates in the binding of AgNPs and ZnO/Ag-NH decoration and thus participates in the formation of reactive species in an aqueous medium, as is the case for cancer cells.

## 1. Introduction

In recent years, carbon nanotubes (CNTs) have garnered increased attention from several research groups as they provide a platform for building complex materials, known as nanobots, that perform intricate functions [1,2]. Among the physical and chemical properties of CNTs, notable ones include electric field modulation [3], strong wear behavior and hardness [4], piezoresistivity [5], and electromagnetic wave absorption [6]. These properties suggest promising uses for CNTs in developing biosensors [7], constructing field-effect transistors [8], micro-energetic applications [9], and environmental remediation [10]. The most common classifications of CNTs include single-walled carbon nanotubes (SWCNTs) and multi-walled carbon nanotubes (MWCNTs) [11]. While it may appear that the primary difference between these two types is that the former has only one layer and the latter has multiple concentric carbon layers, the distinctions are more significant. For example, the addition of layers in CNTs influences differences in thermal conductivity [12], fluid dynamics [13], the density of magneto-hydrodynamic (MHD) electrical flow [14], and biological responses [15]. In particular, MWCNTs have demonstrated a high capacity to bind with organic and inorganic molecules for functionalization [16,17,18]. Thus, several research groups have been investigating functionalization strategies of MWCNTs to take advantage of these structures as tunable platforms [19,20]. Among the functionalization processes, understanding the role of carbon binding in the formation of complex compounds is a crucial aim that allows refined applications toward the highest efficiency [21], which could be particularly useful in medical, environmental, or energy applications [2,22,23]. Charge transfer in solid-state materials involves the movement of electrons and holes, influenced by the electronic structure of the materials, their energy bands, their impurities (doping), and the interfaces formed between different materials in heterostructures [24]. Applications such as water treatment, energy storage, and cancer treatment require compounds with a strong capability for charge transfer due to its role in the formation of a high density of reactive species [25]. Particularly, in carbon-based heterostructures, different materials are combined, each with unique electronic properties. This combination can create interfaces where charge transfer can be significantly enhanced or manipulated due to the differences in energy levels and electron affinity between the materials [26]. There have been efforts to combine MWCNTs with other elements such as fluorine [27,28], where, particularly, the fluorination process has been studied, finding an implication on the atomic distance between carbon atoms due to the chemical binding. Thus, the chemical implications of atom incorporation in MWCNTs are a relevant topic to explore.

This research represents a contribution to understanding the chemical nature of carbon binding with metal and metal oxide nano-heterostructures. MWCNTs were decorated with AgNPs and ZnO/Ag nano-heterostructures using an accessible chemical co-precipitation method assisted with ultrasonic radiation. The chemical and physical properties of the decorated MWCNTs were studied in detail with X-ray photoelectron spectroscopy and correlated with the reactive species in an aqueous medium excited with UV radiation. The relationship between the hybridization of carbon atoms and the binding of ZnO/Ag nano-heterostructures in the generation of reactive species is discussed.

## 2. Materials and Methods

### 2.1. Materials and Reagents

The synthesis process utilized deionized water (resistivity of 18 MΩ, SIGMA, St. Louis, MO, USA SKU: W4502), zinc acetate (Zn(CH_3_COO)_2_·2H_2_O, SIGMA, USA SKU: 1724703), sodium hydroxide (NaOH, SIGMA, USA SKU: S8045), multi-walled carbon nanotubes (MWCNTs, SIGMA SKU: 901019), silver nitrate (AgNO_3_, SIGMA, USA SKU: 1612605), and ethanol (C_2_H_5_OH, SIGMA, USA SKU: NIST2899A). All chemical reagents were used as received, without any additional purification steps.

### 2.2. Synthesis of MWCNT/ZnO

For the synthesis of the MWCNT/ZnO heterostructure, a 100 mL aqueous solution of zinc acetate with a concentration of 0.1 M was initially prepared. Subsequently, 0.22 g of multi-walled carbon nanotubes (MWCNTs) were uniformly dispersed within this solution using an ultrasonic bath for 30 min. Following sonication, sodium hydroxide (NaOH) solution was incrementally added until the mixture achieved a neutral pH. The mixture was then subjected to magnetic stirring for 20 min into an ultrasonic bath to ensure thorough homogenization. NaOH not only adjusted the pH but also acted as a catalyst for the synthesis of ZnO nanoparticles. Post stirring, the mixture underwent centrifugation at 10,000 rpm for 12 min and was dried under a nitrogen flux at 70 °C for 6 h. The final step involved calcining the sample at 450 °C for 4 h in an atmosphere containing 21 wt.% oxygen. This protocol was replicated for separated experiments with agitation periods set from 20 min to 60 min.

### 2.3. Synthesis of AgNPs on MWCNT/ZnO Heterostructure

Once the MWCNT/ZnO heterostructures were obtained, Ag nanoparticles (AgNPs) were attached to the surface of sample. Thus, the heterostructure was synthesized by dispersing 1 g of MWCNT/ZnO in 100 mL of ethanol, followed by 30 min of ultrasonic sonication to achieve an even distribution. Silver nitrate (AgNO_3_), dissolved in 50 mL of ethanol to form a 0.05 M solution (0.4226 g of AgNO_3_), was then added dropwise to the MWCNT/ZnO–ethanol solution. A subsequent 30 min ultrasonic bath ensured the even coating of Ag nanoparticles on the MWCNT/ZnO heterostructure. The solvent was then removed via vacuum drying at 70 °C for 8 h, and the resultant product was calcined at 450 °C for 15 min under a nitrogen flow rate of 20 L/min, effectively securing the Ag nanoparticles to the MWCNT/ZnO heterostructure. This protocol was replicated for separated experiments with different concentration percentages (12% and 24%) of AgNO_3_ in the synthesis reaction.

### 2.4. Physical and Chemical Characterization

The structural characterization of the MWCNT-based heterostructures was conducted using X-ray diffraction (XRD) in a Bragg–Brentano configuration, employing a Panalytical X’pert diffractometer with a Cu-kα radiation source (λ = 1.5418 Å). The diffractograms were obtained through incremental steps of 0.01° across the 2θ range, facilitating the detailed analysis of crystallographic properties. Morphological examination of the samples was performed using a Schottky field emission scanning electron microscope (FESEM) in an AURIGA setup. This setup was equipped with both secondary (SE) and backscattering electrons (BSE) detectors, allowing for comprehensive surface and compositional analysis. Imaging conditions were standardized at a 2 kV acceleration voltage with an 8 mm working distance to optimize resolution and contrast. Furthermore, elemental composition was elucidated using energy-dispersive X-ray spectroscopy (EDS) integrated with the AURIGA microscope, providing a quantitative analysis of the elemental distribution within the heterostructures. Additionally, high-resolution imaging was carried out using a scanning transmission electron microscope (STEM, JEM-ARM200F) in the high-resolution (HRSTEM) mode. This technique offered a more detailed view of the nanostructures, enabling the observation of atomic arrangements and defects. The STEM images were processed and analyzed with Digital Micrograph software (Gatan, USA), enhancing the visualization of structural details. Vibrational properties of the heterostructures were investigated using Raman spectroscopy, utilizing a LabRam HR800 (Horiba Jovin Yvon) spectrometer with a coupled BX41 OLYMPUS microscope and a 633 nm excitation laser. This analysis provided insight into the vibrational modes of the materials, indicative of chemical bonding and structural integrity. Finally, X-ray photoelectron spectroscopy (XPS) analysis was conducted using a Thermo Fisher Scientific K-Alpha photoelectron spectrometer equipped with an Al-kα monochromatic X-ray source, both survey spectra and high-resolution spectra were recorded using pass energy values of 0.5 and 0.01 eV, respectively. Gaussian fitting was employed to fit the high-resolution spectra of carbon, oxygen, and silver. Thus, background subtraction and spectra deconvolution were performed using the algorithms available in the OriginLab program. This technique was pivotal in studying the surface chemistry and electronic transitions of the heterostructures, offering a precise understanding of the electronic environment and the nature of the chemical bonds present. Together, these analytical techniques provided a comprehensive characterization of the MWCNT-based heterostructures, laying the groundwork for understanding their structural, morphological, compositional, vibrational, and electronic properties in the context of this study.

### 2.5. Evaluation of the Reactive Species Generation

In this study, the density of reactive species generated by the effect of the samples was measured indirectly. Thus, the production density of reactive species due to four different samples was analyzed: (1) MWCNT, (2) MWCNT/ZnO, (3) MWCNT decorated with AgNPs, and (4) MWCNT/ZnO with AuNPs (MWCNT/ZnO/Ag). For the experiments, the samples were immersed in a quartz cell containing 3.5 mL of an MB aqueous solution (concentration: 25 μM). These setups were then exposed to UV radiation using a 254 nm UV lamp with a power of 15 W. The irradiation duration was set to 180 min, with the absorbance spectra of the MB solution being recorded every 30 min. This analysis was conducted using a UV–Vis spectrometer (i3 model, Hanon Instruments Co., Ltd., Jinan, China), spanning a wavelength range from 200 nm to 1100 nm. The system utilized both white and deuterium ultraviolet light lamps to facilitate comprehensive spectral measurements. Additionally, the adsorption capability of each sample was determined by exposing 3.5 mL of the MB solution (25 μM) to the samples in darkness for 180 min. The absorbance spectra were similarly monitored at 30 min intervals to assess MB adsorption in the absence of light, thereby differentiating between photocatalytic degradation and adsorptive removal of MB. The degradation rate was linked to the density of reactive species generated, based on the known mechanism for MB degradation where the •OH and •O_2_ reactive species are generated [27,28].

## 3. Results and Discussion

### 3.1. Formation of MWCNT/ZnO/Ag Heterostructure

**Figure 1:**
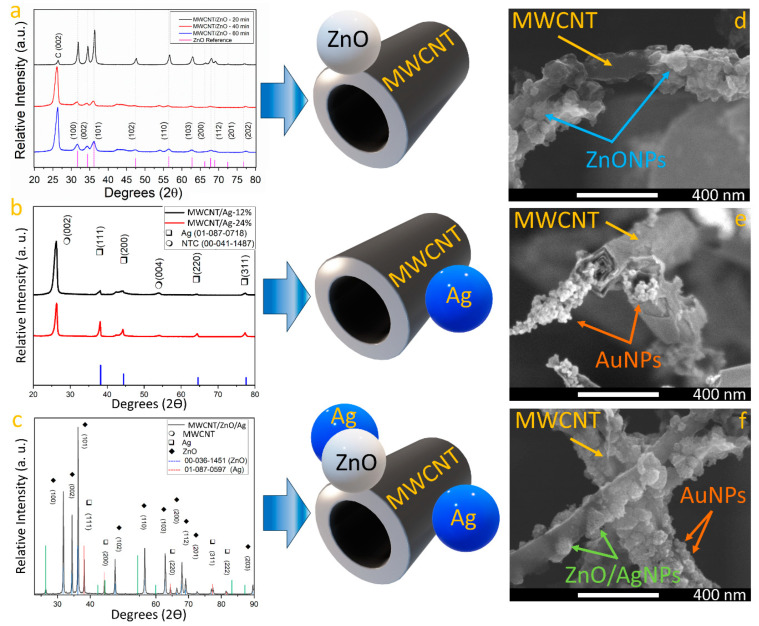
XRD pattern of MWCNT (**a**) across exploration of ZnONPs’ incorporation and (**b**) across exploration of AgNPs’ incorporation, and (**c**) Ag/MWCNT/ZnO/Ag heterostructure. FESEM image of (**d**) MWCNT with ZnO nanoparticles, (**e**) MWCNT with Ag nanoparticles, and (**f**) AgNPs on MWCNT/ZnO heterostructure.

Figure 1a shows the comparison of XRD pattern across time for synthesis in the MWCNT with ZnO. XRD results confirmed the formation of a wurtzite ZnO phase due to the presence of diffraction peaks at 31.82°, 34.47°, 36.30°, 47.45°, 56.46°, 62.76°, 66.21°, 67.805°, and 76.786° degrees correspond well with the (100), (002), (101), (102), (110), (103), (200), (112), (201), and (202) planes of the wurtzite ZnO [29], respectively (reference: 01-079-0207). As the synthesis time increases, the full width at half maximum (FWHM) of ZnO phase-related diffraction peaks is reduced, which suggests that crystallite size decreases with longer synthesis times [30]. As the carbon diffraction peak at the (002) plane of graphite [31] intersects across synthesis time, it is suggested that extended synthesis periods promote the formation of ZnO nanoparticles in the sample. This idea was corroborated with an FESEM image of the sample (see Figure 1d) where a carbon nanotube can be seen with ZnO nanoparticles attached on the complete surface. On the other hand, Figure 1b shows the Ag incorporation to the MWCNT samples across AgNO_3_ concentration in the synthesis process. The diffraction peaks observed at 26.31° and 54.54° correspond to the (002) and (004) planes of graphite [31] (reference: 00-04-1487), which are related to MWCNTs. Moreover, the silver phase was identified by locating the peaks at 38.11°, 44.27°, 64.42°, and 77.47°, aligning with the (111), (200), (220), and (311) planes of cubic Ag, respectively. It was observed that when AgNO_3_ concentrations are reduced in the synthesis procedure, the crystallite size is reduced. An FESEM image of a sample synthesized with 12% of AgNO_3_ concentration is shown in Figure 1e, where AgNPs with a diameter of 21 nm ± 3.2 nm can be seen attached to the MWCNT’s surface. Figure 1c presents the XRD pattern of the Ag/MWCNT/ZnO/Ag sample. The analysis verifies the presence of the three intended materials: MWCNTs, ZnO, and Ag. Zinc oxide manifests in the hexagonal wurtzite phase, with peaks at 31.77°, 34.42°, 36.25°, 47.53°, 56.60°, 62.86°, 66.38°, 67.96°, 69.10°, and 89.60°, corresponding to the (100), (002), (101), (102), (110), (103), (200), (112), (201), and (203) planes, respectively. Regarding silver, peaks noted at 38.11°, 44.29°, 64.44°, 77.39°, and 81.54° are consistent with the (111), (200), (220), (311), and (222) planes, respectively, illustrating the formation of a cubic phase. This comprehensive XRD analysis underscores the successful synthesis and integration of MWCNTs, ZnO, and Ag within the heterostructure, offering valuable insights into their structural characteristics and potential for advanced applications. Field emission scanning electron microscopy (FESEM) images (captured using the secondary electrons detector) of MWCNT/ZnO, MWCNT/Ag, and Ag/MWCNT/ZnO/Ag heterostructures are presented in Figure 1d, Figure 1e, and Figure 1f, respectively. In Figure 1d, zinc oxide nanoparticles are observed adhering to the external surfaces of carbon nanotubes, displaying a mix of crystal sizes characteristic of ZnO. Upon closer examination, the nanotubes are found to be uniformly coated with semispherical nanoparticles, demonstrating a significant density of ZnO nanoparticles with sizes below 80 nm. The presence of Zn, O, and C was corroborated by energy-dispersive X-ray spectroscopy (EDS) analyses (Figure S1). For the MWCNT/Ag heterostructure, a sample synthesized with a 12% AgNO_3_ concentration was chosen (Figure 1e), revealing nanoparticles smaller than those of ZnO, attached to the MWCNTs with diameters below 40 nm. This difference in nanoparticle size between ZnO and Ag could be attributed to their differences in electronegativity [27,28]. The presence of Ag and C in the EDS spectra (Figure S1) was confirmed, with a quantified silver concentration of 10.2 at.% within the analyzed area. Upon obtaining the Ag/MWCNT/ZnO/Ag heterostructure, FESEM characterization (Figure 1f) disclosed nanoparticles with a spheric-like shape attached to the carbon walls. Although the image was acquired using a secondary electron detector, the contrast variation among nanoparticles suggests the existence of multiphase nanoparticles [31]. Consequently, a backscattered electron detector was utilized to capture an image that verified the presence of different phases (Figure S2), likely due to the coexistence of Ag and ZnO within the sample [31,32]. EDS analysis (Figure S1) definitively confirmed the presence of zinc, carbon, oxygen, and silver, with no extraneous elements detected, indicating the methodology’s efficacy in minimizing contamination in the final samples.

**Figure 2:**
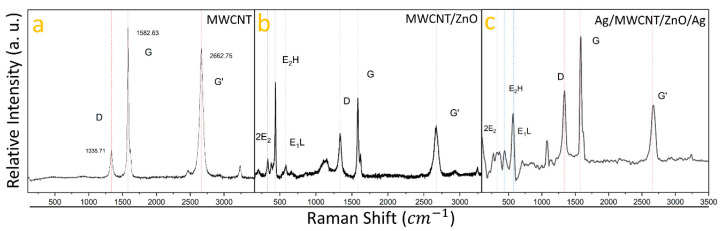
Raman spectral shifts of multi-walled carbon nanotubes (MWCNTs) and their evolution through the incorporation of nanoparticles and heterostructure formation: (**a**) shows the initial Raman spectrum of pristine MWCNTs; (**b**) reveals the spectral changes following the incorporation of zinc oxide nanoparticles (ZnONPs); and (**c**) displays the spectrum for the final Ag/MWCNT/ZnO/Ag heterostructure, highlighting the composite’s vibrational characteristics.

Raman spectroscopy, utilizing a 633 nm excitation source, was employed to investigate the vibrational modes within the heterostructures, as depicted in Figure 2. The Raman spectrum of pure MWCNTs (Figure 2a) reveals peaks placed at 1335.71 cm^−1^, 1582.63 cm^−1^, and 2662.75 cm^−1^, corresponding to the D, G, and G’ bands, confirming the presence of multi-walled carbon nanotubes (MWCNTs) [33]. The MWCNT/ZnO sample’s spectrum (Figure 2b) displays vibrational modes at 330 cm^−1^, 437 cm^−1^, and 579 cm^−1^, characteristic of the 2E2, E2 High, and E1 Low modes of the wurtzite ZnO phase [34], alongside the MWCNT’s D, G, and G’ bands at 1335.71 cm^−1^, 1582.63 cm^−1^, and 2669.84 cm^−1^. The Ag/MWCNT/ZnO/Ag heterostructure spectrum (Figure 2c) exhibits peaks for both ZnO and MWCNTs, with MWCNT peaks at 1338.96 cm^−1^, 1581.59 cm^−1^, and 2667.18 cm^−1^, closely aligning with the carbon D, G, and G’ bands. ZnO vibrational modes are shifted to 328.06 cm^−1^, 442.96 cm^−1^, and 565.72 cm^−1^. A slight shift in the MWCNTs’ D, G, and G’ bands and ZnO peaks indicates that Ag nanoparticles (AgNPs) may be adhering to both MWCNTs and ZnO nanoparticles, subtly altering their vibrational modes, as has been reported in FTIR spectroscopy [35], suggesting an interaction between ZnO, MWCNTs, and Ag that impacts their vibrational properties.

**Figure 3:**
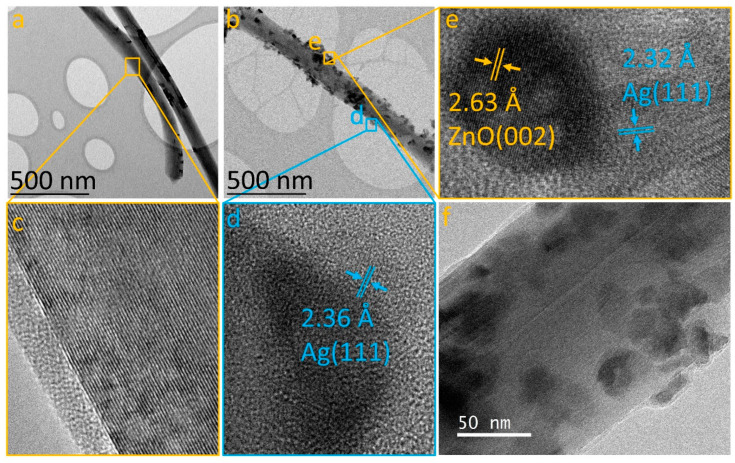
TEM and HRTEM images: (**a**) pristine MWCNT; (**b**) Ag/MWCNT/ZnO/Ag heterostructure; (**c**) detailed MWCNT; (**d**) Ag nanoparticles; (**e**) ZnO/Ag interface; and (**f**) zoomed-in view of the Ag/MWCNT/ZnO/Ag composite.

The heterostructures were analyzed using transmission electron microscopy (TEM) in both low-resolution and high-resolution modes, to elucidate the incorporation of nanoparticles on the MWCNT surface. Figure 3a displays a TEM image of an individual MWCNT, showing a clean surface devoid of additional materials (refer to Figure 3c). Following the bifunctionalization process for the Ag/MWCNT/ZnO/Ag sample, Figure 3b reveals the presence of nanoparticles adhered to the nanotube surfaces, indicating successful heterostructure formation. Two distinct types of nanoparticles are observed: (1) smaller, triangular-shaped particles and (2) larger, spherical particles. High-resolution TEM (HRTEM) imaging (Figure 3d) of each nanoparticle type reveals an interplanar distance of 2.36 Å for the triangular nanoparticles, matching the (111) plane of silver with a face-centered cubic (fcc) structure [36]. Conversely, the spherical nanoparticles exhibit a multiphase structure, as indicated by two different interplanar distances: 2.63 Å, aligning with the (002) plane of hexagonal ZnO [34,37], and 2.32 Å, corresponding to Ag fcc [36]. This suggests that the functionalization process initially forms a ZnO/MWCNT heterostructure, subsequently incorporating Ag onto both the ZnO nanoparticles and MWCNT surfaces, resulting in the formation of the Ag/MWCNT/ZnO/Ag heterostructure. This proposed methodology effectively disperses ZnONPs and AgNPs on the MWCNT surface, addressing a significant challenge in nanotechnology applications [38].

### 3.2. Charge Transfer Process in AgNPs on MWCNT/ZnO Heterostructure

**Figure 4:**
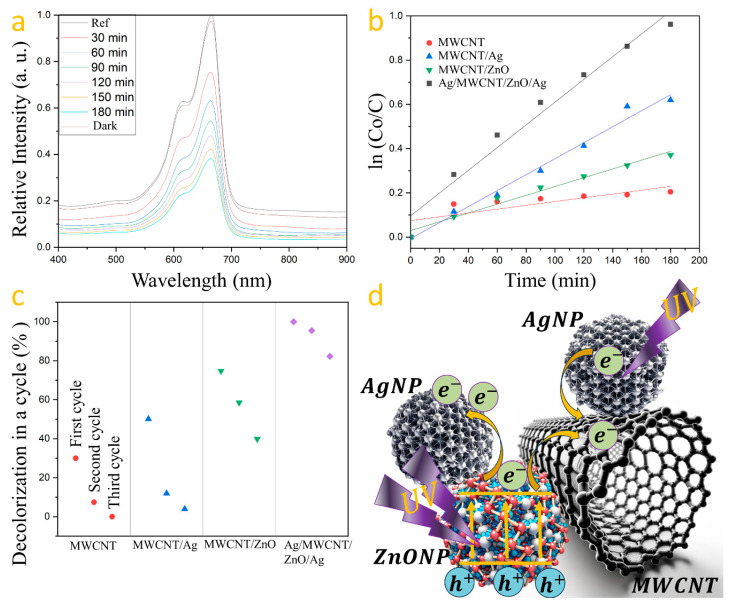
(**a**) MB degradation by Ag/MWCNT/ZnO/Ag, (**b**) MB photodegradation kinetics, (**c**) sample repeatability across degradation cycles, and (**d**) a schematic of the charge transfer mechanism within Ag/MWCNT/ZnO/Ag.

Photodegradation tests were performed on each sample, containing 1 g/L of photocatalyst, under UV light for 180 min. The photocatalysts’ repeatability was assessed over three cycles, with samples washed with deionized water and dried at 80 °C for 3 h after each cycle. The testing solution, made with 50 mL of deionized water and 0.5 mg of MB, was magnetically stirred for 10 min. After setting aside 5 mL of this solution in darkness for 30 min, the photocatalyst was added, followed by UV irradiation for 180 min. Samples of 3 mL were taken at 30 min intervals for analysis. This method was applied to all prepared photocatalysts. Methylene blue’s absorption spectrum, featuring a primary band at 665 nm and a secondary at 616 nm, was analyzed with the 665 nm band used to measure dye degradation [39]. The degradation experiment was conducted for each synthesized photocatalyst, measuring the maximum absorption at the initial time (0 min) as 1.3375 and at 180 min as 0.5109. The percentage of degradation was determined using the following formula: Degradation = (1 − Abs180 min)/Abs0 min × 100, as it is computed in the literature [40]. This calculation was applied to assess the efficiency of each photocatalyst. The resulting degradation percentages for each photocatalyst are presented in Table 1. The reaction kinetics serve as a metric to evaluate the speed of methylene blue degradation, calculated using the formula Ln (Co/C) = kt [41], where k is the rate constant, Co is the initial concentration, and C is the concentration at time t. The MWCNT/ZnO heterostructure with AgNPs’ photocatalyst demonstrated superior degradation efficiency of methylene blue (Figure 4a) as can be seen when the k constant is compared with that computed from the other samples (see Table 1).

Figure 4b compares the kinetic profiles for MB degradation by each photocatalyst. Reproducibility tests over three cycles showed a decline in degradation efficiency: carbon nanotubes from 18.5% to 0.8%, Ag-coated nanotubes from 30.96% to 2.42%, and ZnO-coated nanotubes from 46.2% to 24.61%. However, the Ag/MWCNT/ZnO/Ag photocatalyst exhibited minimal efficiency loss, highlighting the synergistic effect of silver and zinc oxide in enhancing photocatalytic activity, with degradation rates decreasing from 61.80% to 58.99% and then to 50.83% over the cycles (Figure 4c). Figure 4d shows the charge transfer mechanism within Ag/MWCNT/ZnO/Ag heterostructures, highlighting the role of silver nanoparticles in augmenting the charge separation efficiency of ZnO. Upon photon absorption by zinc oxide, electrons are propelled from the valence to the conduction band. The disparity in work functions—5.2 eV for ZnO and 4.6 eV for silver [42]—facilitates the migration of photoinduced electrons towards silver, achieving a balance at the Fermi level [43,44]. Silver nanoparticles act as reservoirs for these electrons, mitigating recombination and thus enhancing photocatalytic performance. Additionally, carbon nanotubes function as conduits for electron transfer to water, generating reactive species and boosting the photocatalytic process. Silver nanoparticles, affixed to the MWCNTs’ surface, act as electron donors, propelled by the photoelectric effect [45], which contributes to the Ag/MWCNT/ZnO/Ag heterostructure’s notable reproducibility and efficiency in methylene blue degradation.

### 3.3. The Chemical Nature of AgNPs on MWCNT/ZnO Heterostructure

**Figure 5:**
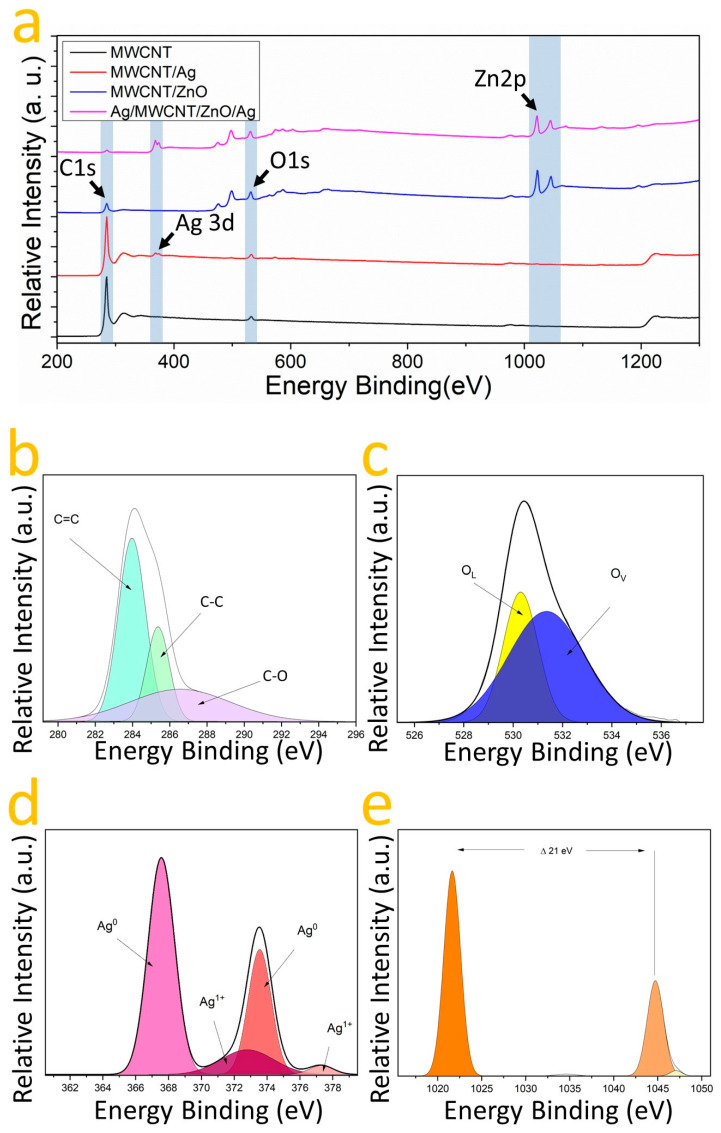
(**a**) showcases XPS spectra: MWCNT (black), MWCNT/Ag (red), MWCNT/ZnO (blue), and AgNPs on MWCNT/ZnO heterostructure (magenta). Gaussian fits for (**b**) C1s, (**c**) O1s, (**d**) Ag3d, and (**e**) Zn2p from AgNPs on MWCNT/ZnO heterostructure are also presented.

X-ray photoelectron spectroscopy (XPS) was utilized to assess the chemical composition and oxidation states of the samples: MWCNT, MWCNT/ZnO, MWCNT/Ag, and MWCNT/ZnO/Ag. This analysis confirmed the presence of carbon and oxygen across all samples, as indicated by the C1s and O1s binding energy (EB) bands (Figure 5a). The deconvolution of the C1s EB band identified two components at 283.62 and 285.62 eV, corresponding to sp^2^ and sp^3^ hybridizations [46], and for oxygen, two components were identified representing oxygen vacancies (Ov) and chemisorbed oxygen (O_C_) at 531.78 and 535.42 eV [47,48]. To corroborate the nature of heterostructure formation and the chemical relationship between the MWCNT, ZnO, and Ag, XPS spectra were fitted using a Gaussian approach. The high-resolution XPS spectra of the elements in the MWCNT/ZnO heterostructure with AgNPs are shown in Figure 5a–e after a Gaussian fit. Thus, Figure 5b shows the C1s oxidation states, identifying C-O_x_, C-C (sp^3^), and C=C (sp^2^) bonds at 286.58, 285.38, and 283.8 eV, respectively, as has been reported in the literature [46,47,48,49,50]. The presence of C-O_x_ species suggests that oxygen is bound to carbon in the MWCNT/ZnO with AgNPs heterostructures [46]. In Figure 5c, the O1s EB band is shown where the spectra unveil an O^2−^ related valence at 530.35 eV linked to the wurtzite structure’s oxygen [39]; moreover, at 531.18 eV, a component indicative of oxygen vacancies in the lattice is visible. In Figure 5e, the analysis of the zinc (Zn) signal reveals two distinct components at 1021.82 eV and 1044.48 eV, corresponding to the Zn2p_1/2_ and Zn2p_3/2_ binding energy (EB) bands, respectively [15]. The observed difference of 21.34 eV between these intensities suggests the presence of zinc oxide (ZnO) [39]. The absence of components other than Zn^2+^ indicates an interaction with oxygen [39]. Figure 5b identifies three carbon components: C=C, C-C, and C=O, at 283.79 eV, 283.81 eV, and 287.17 eV, indicative of sp^2^ and sp^3^ hybridizations and oxygen interaction [46,47,48,49,50,51]. For oxygen, two peaks at 531.14 eV and 532.10 eV correspond to O^2−^ valence and oxygen vacancies, respectively. Figure 5d focuses on silver, showing components at 367.58 and 373.55 eV for the Ag3d_3/2_ and Ag3d_5/2_ EB bands, a 6 eV gap confirming metallic silver’s presence [51,52]. Deconvolution identifies two oxidation states, with 72% Ag0 and 28% in an Ag^1+^ state. A notable shift in the Ag^1+^ valences toward higher energy suggests the formation of silver oxide (AgO) [53], likely due to silver interacting with zinc oxide [50]. These observations, combined with Raman spectroscopy, confirm the interaction between zinc oxide and silver within the heterostructure. Additionally, the coexistence of bonds with carbon and the presence of silver in the Ag^0^ valence state hints at the likelihood of both Ag and ZnO/Ag nanoparticles coating the nanotubes. This multifaceted interaction underscores the complexity and innovative nature of the AgNPs on the MWCNT/ZnO heterostructure.

## 4. Conclusions

Based on the comprehensive analysis provided throughout this work, we conclude that the AgNPs on the MWCNT/ZnO heterostructure exhibit enhanced photocatalytic activity for the degradation of methylene blue (MB) under UV irradiation. The integration of silver (Ag) and zinc oxide (ZnO) nanoparticles onto the surface of multi-walled carbon nanotubes (MWCNTs) not only facilitates improved charge separation efficiency but also significantly boosts the overall photocatalytic performance compared to the individual components. X-ray diffraction (XRD) and X-ray photoelectron spectroscopy (XPS) analyses confirmed the successful formation of the heterostructures, revealing the presence of ZnO in its hexagonal wurtzite phase and metallic silver nanoparticles, both of which are crucial for the observed photocatalytic activity. Field emission scanning electron microscopy (FESEM) and transmission electron microscopy (TEM) imaging provided further evidence of the nanoparticles’ uniform distribution and attachment to the MWCNTs, suggesting a well-defined heterostructure. Raman spectroscopy analysis indicated no significant alteration in the vibrational modes of carbon, implying that the incorporation of Ag and ZnO does not detrimentally affect the structural integrity of the MWCNTs. Instead, it suggests that the presence of AgNPs and ZnO/AgNPs likely enhances the charge transfer mechanisms within the heterostructure, as supported by the photodegradation kinetics and repeatability experiments. The degradation experiments demonstrated that the AgNPs on the MWCNT/ZnO heterostructure result in a higher rate of MB degradation compared to the other configurations tested, with notable reproducibility across multiple cycles. This indicates not only the robustness of the heterostructure but also its potential for practical applications in water treatment and environmental remediation.

## Figures and Tables

**Table 1:** Comparison of degradation values at 180 min and k reaction rate constants.

Sample	MB Degradation (%)	k
MWCNT	18.5	0.0013
MWCNT/Ag	30.96	0.0020
MWCNT/ZnO	46.2	0.0034
Ag/MWCNT/ZnO/Ag	61.80	0.0053

## Data Availability

All data presented in this work are available upon request.

## Supplementary Materials

The following supporting information can be downloaded at: https://www.mdpi.com/article/10.3390/nano14181517/s1, Figure S1: EDS spectra of (**a**) MWCNT/Ag and (**b**) Ag/MWCNT/ZnO/Ag heterostructures; Figure S2: FESEM recorded with backscattered electron detector of (**a**) MWCNT/Ag and (**b**) Ag/MWCNT/ZnO/Ag heterostructures.
