# Supplementary material for: Tuning the Charge Transfer in MWCNTs via the Incorporation of ZnONPs and AgNPs: The Role of Carbon Binding with ZnO/Ag Heterostructures in Reactive Species Formation
Source: Nanomaterials (Basel). 2024 Sep 18;14(18):1517. doi: 10.3390/nano14181517 (PMC11434832; doi:10.3390/nano14181517)
Supplement: Supplementary file 1 [file nanomaterials-14-01517-s001.zip › nanomaterials-3078651-supplementary.pdf]

Supporting Information

Tuning the charge transfer in MWCNT by the incorporation of ZnONPs and AgNPs: The role of carbon binding with ZnO/Ag heterostructures in the reactive species formation

I. Gamiño-Barocio [1], E.F. Vázquez-Vázquez [2], Y. M. Hernández-Rodríguez [1 *], O. E. Cigarroa-Mayorga [1 *]

[1] Dept. Advanced Technologies, UPIITA-Instituto Politécnico Nacional, Av. IPN 2580, C.P. 07340, CDMX, Me-xico.

[2] CINVESTAV-Instituto Politécnico Nacional, Av. IPN, 2508, C.P 07360 CDMX, México.

[*] Correspondence: yazmin.hernandez@cinvestav.mx (YMHR), ocigarroam@ipn.mx (OECM)

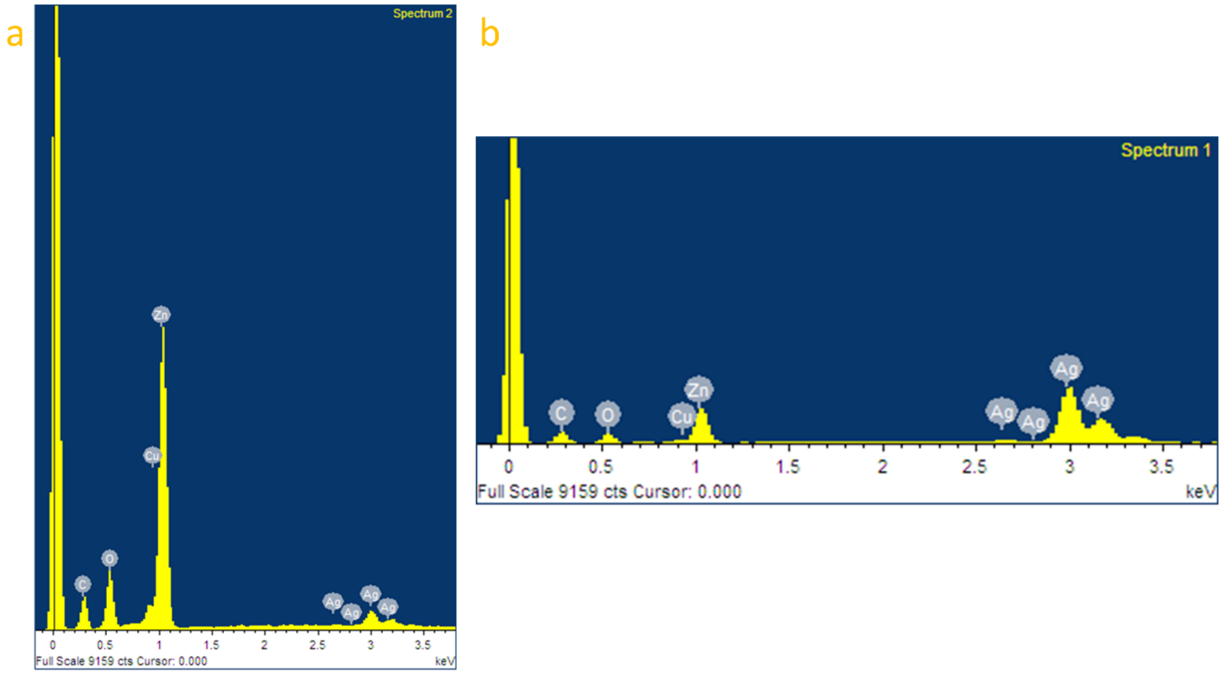

Figure S1. EDS spectra of (**a**) MWCNT/Ag, and (**b**) Ag/MWCNT/ZnO/Ag heterostructures.

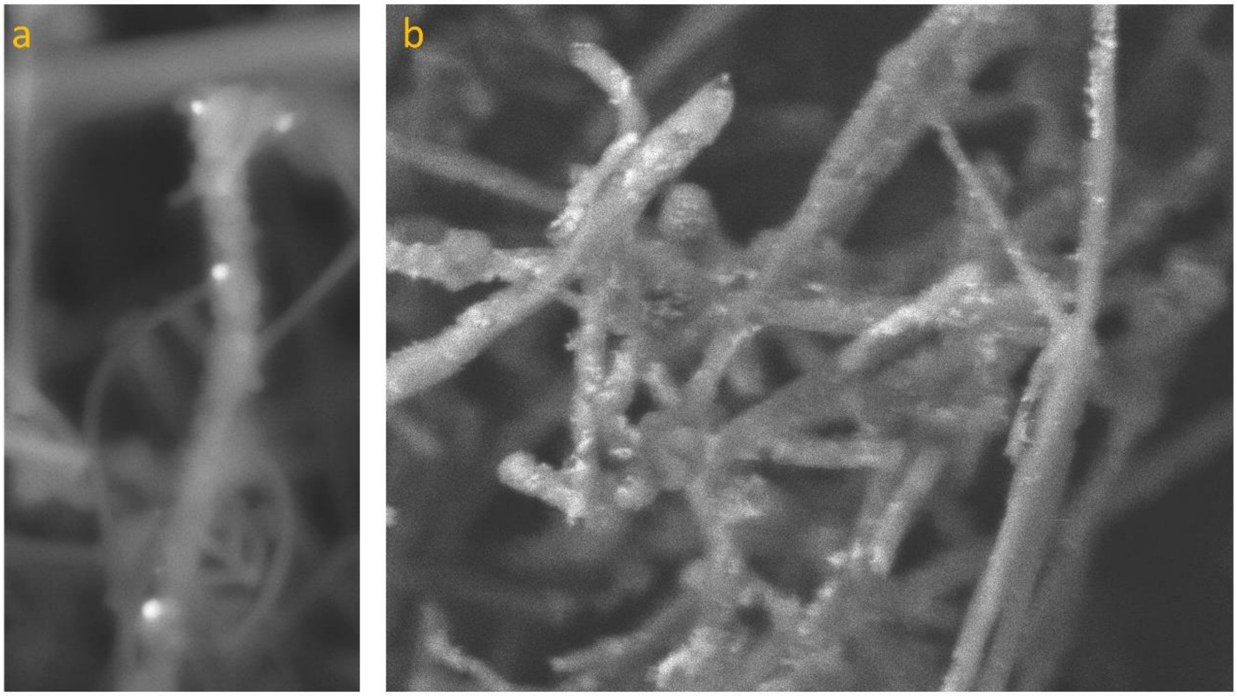

Figure S2. FESEM recorded by backscattered electron detector of (**a**) MWCNT/Ag, and (**b**) Ag/MWCNT/ZnO/Ag heterostructures.
